# Environmental Spread of Antibiotic Resistance

**DOI:** 10.3390/antibiotics10060640

**Published:** 2021-05-27

**Authors:** Nicholas Skandalis, Marlène Maeusli, Dimitris Papafotis, Sarah Miller, Bosul Lee, Ioannis Theologidis, Brian Luna

**Affiliations:** 1 Department of Medicine, Keck School of Medicine at USC, Los Angeles, CA 90033, USA; skandali@usc.edu (N.S.); maeusli@usc.edu (M.M.); 2 Department of Molecular Microbiology and Immunology, Keck School of Medicine at USC, 1441 Eastlake Ave, NTT 6419, Los Angeles, CA 90033, USA; smiller6@usc.edu (S.M.); leebosul@gmail.com (B.L.); 3 Department of Biology, National and Kapodistrian University of Athens, 157 72 Athens, Greece; jimpa7@gmail.com (D.P.); ioannis_theologidis@imbb.forth.gr (I.T.)

**Keywords:** antibiotic resistance, horizontal gene transfer, one health, microbiology

## Abstract

Antibiotic resistance represents a global health concern. Soil, water, livestock and plant foods are directly or indirectly exposed to antibiotics due to their agricultural use or contamination. This selective pressure has acted synergistically to bacterial competition in nature to breed antibiotic-resistant (AR) bacteria. Research over the past few decades has focused on the emergence of AR pathogens in food products that can cause disease outbreaks and the spread of antibiotic resistance genes (ARGs), but One Health approaches have lately expanded the focus to include commensal bacteria as ARG donors. Despite the attempts of national and international authorities of developed and developing countries to reduce the over-prescription of antibiotics to humans and the use of antibiotics as livestock growth promoters, the selective flow of antibiotic resistance transmission from the environment to the clinic (and vice-versa) is increasing. This review focuses on the mechanisms of ARG transmission and the hotspots of antibiotic contamination resulting in the subsequent emergence of ARGs. It follows the transmission of ARGs from farm to plant and animal food products and provides examples of the impact of ARG flow to clinical settings. Understudied and emerging antibiotic resistance selection determinants, such as heavy metal and biocide contamination, are also discussed here.

## 1. Introduction

Antibiotic-resistant (AR) bacteria impose a significant burden on healthcare. In 2017, the Centers for Disease Control (CDC) estimated that there were 2.8 million infections and more than 35,000 deaths in the U.S. due to infections caused by AR bacteria [1]. These estimates are nearly double the previous estimates that were published in 2013 [1,2].

It has been estimated that 20% of these infections are attributable to agricultural antibiotic usage rather than clinical treatment [3]. Foodborne antibiotic-resistant bacteria can survive the harsh conditions of the gastrointestinal tract [4]. Foodborne pathogens can cause acute illness, or they can asymptomatically persist in the gut microbiome as a reservoir for multidrug-resistant, opportunistic, extraintestinal infections [5,6,7,8].

Wastewater also contributes to AR; antibiotics persist through the wastewater treatment processes [9]. Effluents end up in receiving waters, while sludge waste is used as fertilizer [10]. All these sources of antibiotics and AR strains sink to surface waters [11,12] and agricultural soil, which readily absorbs them [13]. Crops become contaminated and serve as vehicles for the transmission of AR bacteria to the food chain [14,15], silently contributing to AR infections [16] or the global burden of illness by directly causing outbreaks of foodborne diseases [17].

The development of AR relies mainly on the prevention of access to drug targets, changes in the structure and protection of antibiotic targets, the direct modification or inactivation of antibiotics, the efflux of antibiotics, and the formation of biofilms [18]. Modifications of antibiotics include enzymatic alteration of the antibiotic by acetylation, phosphorylation and adenylation, and using enzyme like aminoglycoside modifying enzymes (AMEs) [19]. Another modification is the destruction of antibiotic molecules, which in the case of b-lactams is facilitated by b-lactamases [20]. Decreased membrane permeability is also an important mechanism of AR, mostly in Gram-negative bacteria. This is because antibiotic targets are often parts of the inner membrane and antibiotics need to go through the outer membrane first. For example, vancomycin is not effective against Gram-negative bacteria because their outer membrane is impermeable to this antibiotic [21]. Bacteria have developed complex machineries (called efflux pumps) that have the ability to extrude a number of antimicrobial compounds [22]. Another method to combat antibiotics is through interference with their target site. One mechanism of interference is target protection, which affects drugs like tetracycline [23] and fluoroquinolones [24]. Other mechanisms are based on direct modification of the target site and include mutations, enzymatic alteration (for example, methylation), replacement, or overproduction of the target [21,25,26]. Such mechanisms are facilitated by two major genetic strategies: mutational resistance and horizontal gene transfer (HGT) [21].

Another way for bacteria to survive exposure to antibiotics is tolerance. Tolerance is described as the ability to survive lethal concentrations of antimicrobial drugs [27]. Such ability is measured by the minimum duration for killing 99% of the population. Tolerance is based on evolution of mechanisms, such as dormancy [27], or persistence of a subpopulation of cells. These persister cells can survive for a much longer time than the rest of the population [28]. Contrary to dormancy, persistence evolves rapidly following frequent exposure to antibiotics in vitro [29].

**Figure 1:**
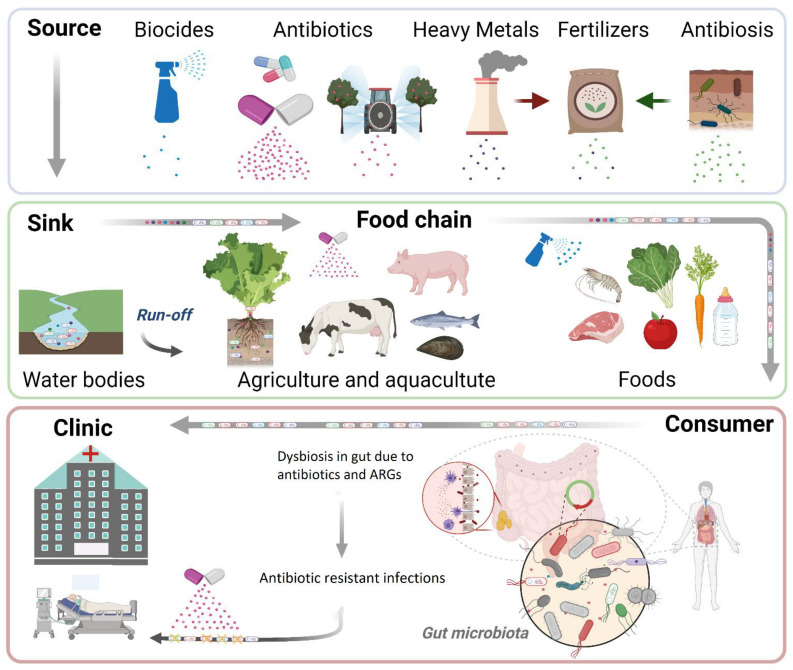
The selective flow of ARGs from the environment to clinic. Clinical, agricultural, and natural antibiotics as well as biocides and heavy metals select for ARGs and contaminate plant, animal, and fish products. Contaminated foods end up into the gastrointestinal tract of humans, where antibiotic resistance emerges from antibiotic presence or is transferred from ARGs to gut microbiota. The endpoints of this antibiotic resistance transmission are human pathogens, which develop AR infections. Created with BioRender.com, accessed on 21 January 2020.

This review focuses on the environmental spread of antibiotic resistance and provides insights further insights into the clinical etiology of AR infections. The selective flow of ARGs is summarized in Figure 1.

## 2. Antibiotics in the Environment

**Natural occurrence of antibiotics.** DNA recovered from 30,000 years old Beringian permafrost sediments indicate that ARGs encoding resistance to beta-lactams, tetracyclines, and glycopeptides predated their clinical use by thousands of years [30]. The coevolution of antibiotics and ARGs contributes to the difficulty of identifying effective natural products against AR bacteria [31].

Ninety percent of antibiotics used in clinics were originally identified from microorganisms [32]. For example, vancomycin, kanamycin, and erythromycin produced by *Streptomyces orientalis*, *Streptomyces kanamyceticus*, and *Saccharopolyspora erythraea*, respectively, were isolated from soil samples [33]. Most of the known antibiotic classes used today come from *Actinomycetes* and especially the genus *Streptomyces*. Those classes include beta-lactams, tetracyclines, macrolides, aminoglycosides, and glycopeptides [34]. It is no surprise that soil systems are abundant reservoirs of naturally occurring antibiotic compounds and anthropogenic contaminants of antibiotics, both of which select for ARGs [35,36,37,38].

Soil actinomycetes, including *Streptomyces*, are a common source of antibiotic compounds. However, the ocean is home to unique actinomycete genera, including *Salinispora* and *Marinispora*. Marine actinomycetes can produce secondary metabolites with antimicrobial activity [35]. For example, coastal water sampling in Southern California has led to the isolation of Marinomycin A, a natural product with antibiotic activity against methicillin-resistant *Staphylococcus aureus* [MRSA] and vancomycin-resistant *Enterococcus faecium* [VREF] [36]. Pestalone is another natural antimicrobial product against MRSA and VREF produced only in the co-culture of a marine fungus, *Pesalotia* sp., with an unidentified marine bacterium [39,40].

**Contribution of agriculture and wastewaters in antibiotic pollution.** The majority of antibacterial agents (including ionophores) purchased in the U.S. are for agricultural use (15.4 million kg, or 80% of the annual total in 2014) [3,41]. Antibiotics from a wide range of classes, including macrolides, lincosamides, sulfonamides, thiamphenicol analogs, and fluoroquinolones have been detected in agroecosystems [42]. Although antibiotics today are used at therapeutic doses to treat existing infections in livestock, they were also often administered prophylactically to prevent illness until 2017 [43]. More importantly, subtherapeutic doses were delivered to livestock over extended periods of time as feed additives to promote growth [43,44].

Another agricultural contributor to the spread of resistance, which has been overlooked, is the use of antibiotics for crop protection [45]. Plant antibiotics have historically accounted for less than 0.5% of total antibiotic use [46]. However, the recent approval for application of streptomycin and oxytetracycline to prevent the spread of citrus canker and citrus greening pandemic diseases led to an 18-fold increase of the agricultural usage of these antibiotics [47]. This regulatory change could result in an unprecedented emergence of ARGs in plant foods. In addition, the combinational use of antibiotics and biopesticides, the latterconsisting of bacterial species that have been selected based on their genetic competence to produce antibiotics [15], resist counter antibiosis [48], and colonize plant niches [49] poses another threat.

Unmetabolized antibiotics found in hospital effluents can be carried into wastewater treatment plants where the removal of antibiotics can be incomplete, ultimately feeding ARGs into the natural aqueous environment [50]. The contribution of wastewater to the spread of antibiotic resistance is supported by AR patterns in wastewater treatment plants that mirror their respective clinical prevalence [9]. Since most of these plants are not designed to completely remove contaminants, antibiotics persist through the wastewater treatment processes. Effluents end up in receiving waters (analyzed below), while sludge waste is used as fertilizer [10]. All of these sources of antibiotics and antibiotic resistance sink to surface waters where antibiotic concentrations in the micrograms per liter range have been reported [11,12]. The antibiotics with the highest concentrations detected in receiving water were trimethoprim, sulfisoxazole, ciprofloxacin, and albendazole [12].

## 3. Emergence of Antibiotic Resistance in the Environment

**ARGs in soil.** Agricultural antibiotics, manure from livestock, and hospital sewage (as well as municipal, agricultural, and aquaculture wastewater) are important sources of antibiotic residues that contaminate soil [51,52,53]. Therefore, soil bacteria act as a reservoir of ARGs [54,55]. Multiple studies have shown a substantial increase in AR nonpathogenic, environmental bacteria. More than 97% of the 123 strains tested were resistant to ciprofloxacin and almost 50% were resistant to erythromycin. Environmental strains carrying ARGs don’t have to necessarily be closely related to human pathogens. Denitrifying bacteria, classified in *Brachymonas*, *Candidatus Competibacter*, *Thiobacillus* and *Steroidobacter* genera, found in the anoxic wastewater treatment process in pig farms are also important hosts of ARGs [56]. *Pseudomonas* is also a dominant genus in the environment that consists of many species, such as the nonpathogens *P. fluorescens* and *P. putida* and the very important clinical pathogen *P. aeruginosa*, which is often associated with multidrug resistance phenotypes [57,58].

Wastewater irrigation can also affect the soil resistome. It has been shown that irrigation with untreated wastewater can increase the amount of multidrug resistant bacteria even after long periods of no irrigation [59]. Dantas et al. isolated multidrug resistant (MDR) soil bacteria that could also grow in the presence of several of the tested antibiotics, suggesting that the soil reservoir contributes to the increasing levels of MDR pathogenic bacteria [60]. This ARG reservoir may serve as a source of ARG transmission between nonpathogenic soil bacteria and human pathogens as previously described by others, but the overall dynamics of this phenomenon have not been associated with clinical practice [54,61].

**ARGs in water bodies.** Urban and coastal water systems can serve as gateways for the dissemination of anthropogenically associated ARGs [62,63]. Antimicrobials and the selection of ARGs occur in beef cattle storage ponds and swine treatment lagoons, [64] but also in water samples collected throughout the Pacific Ocean [37]. AR bacteria can also be transferred between locations by birds or other animal species [65,66].

Studies in the Antarctic have also provided an important model to study the dissemination of resistance genes in aqueous environments with minimal human interference [38,67,68]. Studies have found clinically relevant ARGs at sampling sites close to field research stations supporting transmission routes of human origin from wastewater plants [38,69]. Hernandez et al. reported ESBL genes *bla*_CTX-M1_ and *bla*_CTX-M15_ in seawater samples collected near Antarctic field stations [38]. Another study also reported ESBL genes (*bla*_CTX-M2_ and *bla*_PER-2_) and “plasmid-mediated AmpC beta-lactamase genes” (*pAMP*_CDHA_, *pAMP*_CFOX_) in nearby freshwater samples [67].

## 4. Co-Selection of ARGs Due to Other Pollutants

Major heavy metal pollutants (such as cadmium, copper, lead, chromium, arsenic, and mercury) are ubiquitous metal pollutants of soil and water due to their presence (as byproducts) in fertilizers, construction materials, and antifouling paints [70,71]. Exposure to heavy metals mainly occurs through the food chain via plant root absorption or direct ingestion via drinking of contaminated groundwater [72]. Recently, the U.S. Congress reported that baby food is tainted with dangerous levels of heavy metals [73]. Heavy metals in excess concentrations can interfere with vital cellular functions and are highly toxic to most organisms [74]. On the contrary, heavy metals are of moderate to high physiological importance for some bacterial species [75]. Bacteria have coevolved resistance mechanisms to heavy metals and antibiotics, based in extra- and intracellular sequestration, enzymatic detoxification, and metal removal [76]. Such resistance mechanisms are thought to converge [77] (based on the co-occurrence of respective resistomes in bacterial genomes [78]) and increase resistance in the absence of antibiotic treatment [79].

Other nonantibiotic antimicrobial compounds that have been observed to coselect with ARGs are biocides [79]. Common uses of biocides include: disinfectants on equipment and surfaces in facilities like farms and hospitals, antiseptics on body surfaces, decontaminants and preservatives in pharmaceuticals, and food [80]. Biocides are used in large quantities; the 2006 market in Europe was estimated at 10–11 billion euros, and it is believed that usage has only increased since then [81]. Their consumption has risen dramatically due to the Covid-19 pandemic [82]. Consequently, it is no surprise that biocides have found their way into the environment. For example, high amounts of triclosan and other biocides have been detected in rivers and wastewater treatment plant (WWTP) effluents. More specifically, 138 g/day of triclosan and 214 g/day of triclocarbon were released into the Savannah River in Georgia (U.S.) from three WWTPs [83]. In a different study conducted in eight WWTPs and the receiving aquatic environment in Thailand, they found high amounts of methylparaben up to 15.2 μg/L in the receiving Chao Phraya River, 8.47 μg/g of triclocarbon in sludge and sediment and 1.20 μg/g of triclosan in fish samples [84].

Bacteria have developed various resistance mechanisms to biocides [81,85], including target alteration [86,87], impermeability [88,89], efflux pumps [90,91], and inactivation of biocides [92,93]. Similar to heavy metal resistance, biocide resistance has the ability to coselect with antibiotics [79] and enhance antibiotic resistance [94]. For example, exposure to benzalkonium chloride increased the microbial community MICs for benzalkonium chloride, ciprofloxacin, tetracycline and penicillin G [95]. Benzalkonium chloride, as well as chlorhexidine digluconate [96], can induce the multidrug efflux pump MexCD-OprJ in *Pseudomonas aeruginosa*, contributing to resistance to fluoroquinolone antibiotics [97]. In a recent study, low level exposure to chlorhexidine digluconate (24.4 μg/L) and triclosan (0.1 mg/L) in *E.coli* have been shown to significantly increase horizontal transfer of mobile AR genetic elements by conjugation [98]. Triclosan exposure can also reduce the susceptibility to clinical antimicrobials, like ciprofloxacin and levofloxacin, in *E. coli* isolates from urine samples [99]. In addition, exposure of *Salmonella enteritidis* to chlorine increased the MIC values eight-fold for tetracycline, nalidixic acid, and chloramphenicol [100]. Information on the actual contribution of biocides to ARG emergence and transmission in the food chain remains scarce [101].

## 5. Transmission of ARGs across the Food Chain

**ARGs in meat, poultry and fish products.** It has been well established that livestock and animal products contribute to the spread of AR bacteria and genes to humans [4,8,102,103,104]. Most antibiotics purchased in the U.S. are for use in agriculture [105]. Livestock are fed these antibiotics, thereby creating a selective pressure favoring ARGs in the animal gut and feces [4,102,103]. In Belgium, about 35% of the *E. coli* strains isolated from live broilers were resistant to third generation cephalosporins, while over 60% of the broilers were found to be carriers of these third generation cephalosporin resistant *E. coli* [CREC]. AR strains can also contaminate meat industry employees. Hog slaughterhouse employees demonstrated similar numbers of *Staphylococcus aureus* isolates in comparison to their family and community members [106]. However, the employees’ isolates were resistant to more antibiotic types suggesting a greater selective pressure originating at the hog plant [106].

Contamination of animal products starts during slaughter and spreads throughout the food supply chain [8,104]. Even if food processing methods are applied in order to kill bacterial cells, dead cells may remain intact or be lysed and release ARGs [107]. The subsequent spread of AR bacteria and their genes can happen in the kitchen during meal preparation and by the incomplete cooking of meat surfaces prior to consumption [4,7]. Hands and cutting boards are known sources of cross contamination with ESBL-producing *E. coli* [108]. Furthermore, the increasing demand of minimally processed or raw fish and meat further contaminates such products with ARGs [109]. Shiga toxin-producing *Escherichia coli* (STEC) serotypes, including O157:H7 strains, were isolated from dairy cows, cull dairy cow feces, cider, salami, human feces, ground beef, bulk tank milk, and bovine feces in media selective for different antibiotics [110]. Extraintestinal pathogenic *E. coli* (ExPEC) and other antibiotic-resistant *E. coli* have been found in poultry, pork, and beef at grocery stores [8]. In a study performed in Austria, resistant *E. coli* isolates were found most often in pork (76%), followed by poultry (63%) and beef (40%) [111]. Similarly, the most predominant *E. coli* ARGs isolated from chicken meat were *tetA* (for tetracycline), *aadA1* (for streptomycin), *ereA* (for erythromycin), *aac-3-IV* (for gentamicin), *cmlA* and *catA1* (for chloramphenicol) [112].

Aquaculture is the fastest growing food production sector representing 47% of global fish production (80 million tons), equating to a $231.6 billion (USD) industry [113]. While the growth and revenue of the aquaculture industry is beneficial for feeding the world’s growing population, it is alarming that antibiotics are frequently used for prophylaxis and metaphylaxis in aquaculture without substantial regulation in the countries producing the most fish [104,114]. One such method includes the application of antibiotics with feed in open aquaculture cages. This method allows for unmetabolized antibiotics in fish excrements and unconsumed excess antibiotics to spread into surrounding water and sediment, particularly in the absence of collection systems [115]. Aquaculture waste is also used as fertilizer for land based agriculture, yet another means of spreading AR bacteria and their genes into human food sources [116]. Market finfish and shellfish can be contaminated with bacteria resistant to clinically important antibiotic classes, including tetracyclines, beta-lactams, aminoglycosides, and quinolones [117,118,119,120].

**ARGs in Produce.** While most scientific focus in agricultural sources of antibiotic resistance has been in livestock and meat, the role of vegetables in the spread of ARGs has been largely overlooked [121,122,123]. Likewise to the previously described AR bacteria transmitted via animal products, resistant bacteria transmitted from plants can also cause acute illness or asymptomatically colonize the gut [124]. Clinically relevant ARGs and bacteria, such as *E. coli*, have also been found on vegetables [7,122,123,125]. Even multidrug resistant strains of *Acinetobacter baumanii*, a pathogen listed under the most urgent threats by the U.S. CDC, have been reported on produce and fruit [121,122,123].

Little is still known about what plant characteristics, human behaviors, and bacterial properties drive the transmission of antibiotic resistance from produce to the mammalian gut microbiome. One possible mechanism may include persister cell populations. Persister cells of *E. coli* O157:H7, the causing agent of foodborne illness, increased in low humidity conditions on lettuce [126]. *Salmonella* persister populations in the gut have been identified as reservoirs for antibiotic resistance plasmids, and they were able to transmit these resistance genes to gut *E. coli* [127]. Agricultural use of antibiotics also drives selection flow of ARGs to produce. The presence of *strAB* genes and streptomycin-resistant genes in plant pathogens, such as *Erwinia amylovora*, *Pseudomonas syringae,* and *Xanthomonas campestris*, preceded the agricultural use of streptomycin. Such genes are thought to be acquired from nonpathogenic epiphytic bacteria colocated on plant hosts under natural antibiotic selection [128].

Further to previous findings, we suggest that ubiquitous bacteria harbor multiple ARGs of clinical importance. *Pseudomonas corrugata*, which acts as an opportunistic pathogen [129], was found to be resistant to cefepime, gentamicin, polymyxin B, and chloramphenicol (Table 1), which are currently used therapeutically for *Pseudomonas aeruginosa* and other human pathogenic Pseudomonads [130]. Similarly, the ubiquitous soil bacterium and opportunist plant pathogen *Pectobacterium carotovorum subsp carotovorum* [131] was found to be resistant to cefepime, gentamycin, and chloramphenicol (Table 1), which are clinically used against pathogenic *Enterobacterales* [130]. Finally, *Bacillus thuringiensis* sbsp. *kurstaki* (which is used commercially as a bioinsecticide [132]) was found to be resistant against ampicillin, penicillin, and erythromycin, which are clinically used against Bacillus pathogens other than B. anthracis [133]. Such findings are clearly suggestive of the acquisition of ARGs due to natural competition of these dominant environmental species with other plant-associated bacteria. ARGs can enter the food chain and ultimately end up in the human gut [127].

Little focus has been placed on directly modeling the mechanisms of transmission from plant foods to the gut microbiome [122,123,124,136] (Figure 1). The gut microbiome can serve as a reservoir of ARGs in asymptomatic human hosts [137]. Previous research by our group has demonstrated that lettuce can serve as a platform for the horizontal gene transfer of antibiotic resistance plasmids from nonpathogenic bacteria harboring mobile ARGs to clinically relevant, pathogenic *E. coli* [61]. Moreover, the challenge of mice by oral gavage of an AR *E. coli* clinical isolate suspended in a lettuce homogenate resulted in asymptomatic colonization of the gut and additionally allowed for the horizontal transfer of resistance to resident *Klebsiella pneumoniae* in the gut [61].

## 6. Clinical Outcomes

In the United States between 2012–2017, there was a decline in the number of cases of multidrug resistant infections of methicillin-resistant *S. aureus* (20.5%), vancomycin-resistant *Enterococcus* (39.2%), carbapenem-resistant *Acinetobacter* spp (32.0%), and MDR *P. aeruginosa* (29.7%) [138]. No trend was observed for the change of carbapenem-resistant Enterobacteriaceae during this time period. However, there was the notable exception of a 53.3% increase in ESBL-producing Enterobacteriaceae from 2012–2017 [138,139].

Predictably, the increase in the frequency of antibiotic resistance has also resulted in increased mortality. In Europe, between 2007–2015, it was found that there was an increase in the estimated number of related infections due to AR *E. coli*, *S. aureus*, *P. aeruginosa*, *K. pneumonaie*, *E. faecalis*, *E. faecium*, and *S. pneumoniae* from 239,238 cases to 602,609 cases [140]. A proportional relationship for the number of cases and the number of attributable deaths was observed for these pathogens, and the greatest number of cases (285,758) and deaths (8750) were observed for third-generation cephalosporin-resistant *E. coli* [140]. Additionally, this resulted in an increase from 11,114 to 27,249 attributable deaths over this same time period [140]. The relative increase in mortality attributed to each of the pathogens studied was variable. The greatest increase in mortality was observed for carbapenem-resistant *K. pneumoniae,* which was attributed to 341 and 2094 deaths in 2007 and 2015, respectively.

Hospitals are a hotspot for the emergence of AR bacteria due to the relative high density of patients with bacterial infections and the use of antibiotics and other antimicrobial disinfectants that may also inadvertently select for increased resistance. Unsurprisingly, surveillance studies often report the presence of AR bacteria on hospital surfaces and also in the water system [141,142,143]. The abundance of AR bacteria and/or resistance conferring genes within the hospital are risks for direct transmission to patients. It has been common practice for hospitals to track antibiotic resistance by isolating and characterizing individual clinical isolates. However, it has been difficult to attribute changes in antibiotic resistance patterns to specific examples of horizontal gene transfer. Most of the evidence regarding transfer of antibiotic resistance from animal foods has been based on the identification of *E. coli*, mostly clones and ARGs that are indistinguishable in both food and human isolates [144]. Recent advances in sequencing technology and whole genome sequencing may now provide the resolution for studying genetic relatedness, which will allow for the real-time monitoring and detection of plasmid transfer dynamics [145,146].

Significantly less data exists for characterizing the horizontal gene transfer of ARGs in a patient. Conjugative transfer of a mupirocin-resistance plasmid has been described between *Staphylococcus epidermidis* to methicillin-resistant *Staphylococcus aureus* in a nursing home resident [147]. Another study was able to show the likely plasmid transfer between *E. coli* and *K. pneumoniae* within a single patient and, additionally, that same plasmid was likely transferred to a second patient [146]. Broad-host Gram-negative plasmids have also been described to transfer the *bla*_KPC_ gene that resulted in the spread of carbapenem resistance among *Citrobacter freundii*, *Enterobacter cloacae*, *Klebsiella aerogenes*, and *Klebsiella pneumoniae* in a transplant patient [148]. In addition to the more simple mechanism of the direct transfer of plasmids from one bacterium to another, plasmid dynamics can be much more complex and require genetic rearrangement involving additional plasmids, thus creating even more complexity [148,149]. Recently, mechanistic models were employed to combine date from 9000 patients and characterize the dissemination routes of a pOXA-48-like carbapenemase-encoding plasmid in a hospital setting over a 2-year period [150].

## 7. Conclusions

Antibiotic resistance continues to be a significant problem. While the mechanics of how genetic information can be transferred from one bacterium to another are generally understood, there remain significant knowledge gaps in how ARGs are trafficked from environmental sources to humans and animals (Figure 1). Little information is available about the inter- and intraspecies transfer of ARGs in vivo. However, recent advances in genomics tools and technologies will allow for real-time monitoring of ARG transfer dynamics. A better understanding of how ARGs are trafficked will allow for improved strategies to mitigate resistance transmission, with the ultimate goal of reducing morbidity and mortality associated with AR infections.

## Figures and Tables

**Table 1:** Susceptibility testing of the plant-pathogenic bacterium *Pseudomonas corrugata*. Methods were used as previously described [134,135]. Interpretive criteria (S: susceptible, I: intermediate, and R: resistant) are based on the Clinical and Laboratory Standards Institute breakpoints. *Pseudomonas corrugata* strain 870 BPIC (Benaki Phytopathological Institute Collection) breakpoints correspond to (a) “other non-Enterobacterales including *Pseudomonas spp.* but excluding *P. aeruginosa*” breakpoints [130] (R1, I1, S1 in red); (b) “*Pseudomonas. aeruginosa* breakpoints [130] (R2, I2, and S2 in blue). *Pectobacterium carotovorum subsp carotovorum* isolate 3412/17 BPIC breakpoints correspond to *“Enterobacterales breakpoints”* [130] (R1, I1, and S1 in black). *Bacillus thuringiensis* sbsp. *kurstaki* strain ABTS-351 (ATCC-SD-1275) breakpoints correspond to “*Bacillus* spp. and related genera (not *B. anthracis*)” breakpoints [135]. AMP: ampicillin, PEN: penicillin, FEP: cefepime, VAN: vancomycin, FOF: fosfomycin, ERY: erythromycin, CLI: clindamycin, GEN: gentamicin, MEM: meropenem, TET: tetracycline, PMB: polymyxin B, CHL: chloramphenicol, CIP: ciprofloxacin, RIF: rifampicin, LCM: lincomycin.

*Pseudomonas corrugata*															
Antibiotic	AMP	PEN	FEP	VAN	FOF	ERY	CLI	GEN	MEM	TET	PMB	CHL	CIP	RIF	LCM
MIC (mg/L)	>32	>64	32	>64	256	>64	>8	16	8	1	>16	32	<0.5	32	>32
Breakpoint			R_1_ ≥ 32 ≤ R_2_					R_1_ ≥ 32 ≤ R_2_	I_1_ = 8 ≤ R_2_	S_1_ ≤ 4	R_2_ ≥ 4	R_1_ ≥ 32	S_1_ > 0.5 = S_2_		
*Pectobacterium carotovorum* sbsp. *carotovorum*															
Antibiotic	AMP	PEN	FEP	VAN	FOF	ERY	CLI	GEN	MEM	TET	PMB	CHL	CIP	RIF	LCM
MIC (mg/L)	>32	>64	8	>32	128	32	>8	32	<1	1	<1	128	<0.5	4	>32
Breakpoint	R ≥ 32		R ≥ 16		R ≥ 256			R ≥ 16	R ≥ 4	R ≥ 16	R ≥ 4	R ≥ 32	R ≥ 1		
*Bacillus thuringiensis* sbsp. *kurstaki*															
Antibiotic	AMP	PEN	FEP	VAN	FOF	ERY	CLI	GEN	MEM	TET	PMB	CHL	CIP	RIF	LCM
MIC (mg/L)	32	16	>64	<4	64	>8	1	<2	<1	<2	>16	<4	<0.5	<0.5	16
Breakpoint	R ≥ 0.5	R ≥ 0.25		S ≤ 4		R ≥ 8	R ≥ 4	R ≥ 16	R ≥ 16	R ≥ 16		R ≥ 32	R ≥ 4	R ≥ 4	
